# An Objective Methodology for the Selection of a Device for Continuous Mobility Assessment

**DOI:** 10.3390/s20226509

**Published:** 2020-11-14

**Authors:** Tecla Bonci, Alison Keogh, Silvia Del Din, Kirsty Scott, Claudia Mazzà, on behalf of the Mobilise-D consortium

**Affiliations:** 1 Department of Mechanical Engineering & INSIGNEO Institute for in Silico Medicine, The University of Sheffield, Sir Frederick Mappin Building, Mappin Street, Sheffield S1 3JD, UK; kscott3@sheffield.ac.uk (K.S.); c.mazza@sheffield.ac.uk (C.M.); 2 School of Public Health, Physiotherapy and Sports Science, University College Dublin, 4 Dublin, Ireland; alison.keogh@insight-centre.org; 3 Translational and Clinical Research Institute, Faculty of Medical Sciences, Newcastle University, Newcastle upon Tyne NE4 5TG, UK; Silvia.Del-Din@newcastle.ac.uk

**Keywords:** wearable technology, real-world assessment, continuous monitoring, healthcare challenges, inertial measurement units, digital mobility outcomes, mobility assessment

## Abstract

Continuous monitoring by wearable technology is ideal for quantifying mobility outcomes in “real-world” conditions. Concurrent factors such as validity, usability, and acceptability of such technology need to be accounted for when choosing a monitoring device. This study proposes a bespoke methodology focused on defining a decision matrix to allow for effective decision making. A weighting system based on responses (*n* = 69) from a purpose-built questionnaire circulated within the IMI Mobilise-D consortium and its external collaborators was established, accounting for respondents’ background and level of expertise in using wearables in clinical practice. Four domains (concurrent validity, CV; human factors, HF; wearability and usability, WU; and data capture process, CP), associated evaluation criteria, and scores were established through literature research and group discussions. While the CV was perceived as the most relevant domain (37%), the others were also considered highly relevant (WU: 30%, HF: 17%, CP: 16%). Respondents (~90%) preferred a hidden fixation and identified the lower back as an ideal sensor location for mobility outcomes. Overall, this study provides a novel, holistic, objective, as well as a standardized approach accounting for complementary aspects that should be considered by professionals and researchers when selecting a solution for continuous mobility monitoring.

## 1. Introduction

Mobility is recognised as one of the vital signs, as reduced mobility, reflected by slower walking speed and its reduction over time, has been associated with greater mortality, morbidity, cognitive decline, dementia, and falls risk [1]. Therefore, walking speed could be used as an outcome to monitor health and function, and evaluate innovative interventions or drug treatments [1,2,3]. Measurement of mobility usually occurs in laboratory or clinical settings [3], where individuals’ mobility capacity (what they can do) is tested under standardised conditions. However, this assessment could be influenced by clinicians’ subjectivity or by patients’ extra effort during short-term examinations [4]. Mobility performance (what they actually do) is instead assessed in the real-world and may show a better discriminative validity, especially in diseases characterised by specific mobility dysfunctions and fluctuations, such as in Parkinsons Disease [5]. Therefore, continuous mobility monitoring could detect, measure, and eventually predict mobility loss linked to a change in speed. This is, in turn, may provide essential information for a personalized treatment [1,3] as well as other adverse clinical events or outcomes. Therefore, a low-cost, easy-to-use, and accurate approach using technology that can operate in “real-world” scenario is essential to complete this aim, and wearable devices are ideal candidates.

A variety of data processing algorithms to estimate digital mobility outcomes (DMOs, e.g., walking speed, cadence, etc.), from either a single or multiple devices, have been proposed and validated [6,7,8,9,10,11,12,13,14,15,16,17,18,19,20,21,22], and the effect of different device locations has also been assessed [23,24,25,26,27,28,29,30,31,32]. Nonetheless, algorithms and associated wearable devices are still far from widespread adoption outside of research labs due to several other limiting factors, such as human factors, wearability, usability, and data capture.

Effective deployment of continuous mobility monitoring is strongly influenced by the perception and acceptability of a wearable device to the user [33] and its wearability and usability [34,35]. However, these aspects have not been widely investigated. Usability of different wearable devices has been assessed in older adults [36,37], patients with chronic obstructive pulmonary disease (COPD) [38,39], adults with chronic diseases [40], and on target populations interested in wearable solutions (e.g., novel vs. experienced users [34]). The data capture process, and the amount of interaction expected from the participant, might also be a limiting factor in adopting wearable devices. For example, to enhance the accuracy of DMOs, some approaches require the subject to perform a given movement before data acquisition (e.g., holding a static posture as in Bugané et al. [7]) or input anthropometric measures [6,21], which can affect the overall experience for both participants and assessors.

While all of the above factors should be considered when selecting a wearable device for continuous mobility assessment, typically, these have only been considered in isolation [30,40,41] or as subsets [27,38,39]. One reason for this is the lack of a structured methodology to combine and objectively evaluate such various factors for a comprehensive assessment of concurrent wearable devices. Among these methodologies, decision matrices, typical of well-established design processes [42], are the most practical and objective tools for a multi-domain evaluation approach in selecting one option from several alternative solutions. Therefore, the primary aim of this study is to design a bespoke decision matrix to assist in selecting the optimal wearable device for continuous mobility monitoring. The study will initially identify the factors to be evaluated and their relevant scoring criteria (i.e., scoring system). The relevant importance of these factors in the overall assessment will then be established considering the perspective of professional and research staff using an ad-hoc questionnaire. This information will then be used to determine the decision matrix, and different practical examples of its use will be provided.

## 2. Materials and Methods

**Figure 1:**
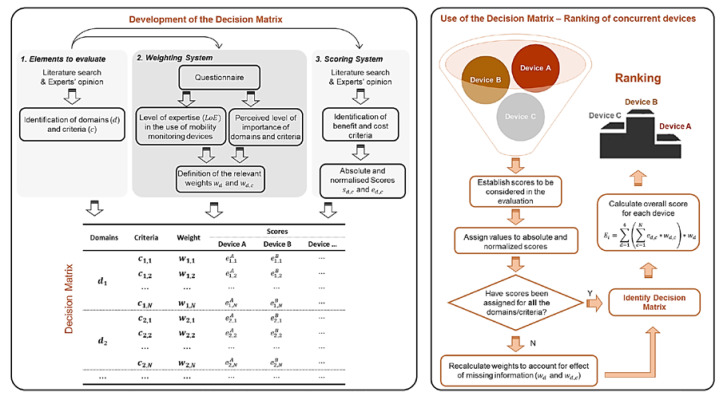
(**Left Panel**) Structure of the procedures required to identify the three elements that compose a decision matrix. LoE = level of experience in the use of wearable devices. (**Right Panel**) Visual representation of the use of the decision matrix for ranking different wearable devices.

A decision matrix (Figure 1) is generally constituted of three main elements: (1) the different elements to be evaluated, (2) a weighting system to establish their relevant importance, and (3) a scoring system to rank various solutions [42]. The following sections describe how these elements were established in this study. A demonstration of how this tool can be used is also provided using data available in the literature.

### 2.1. Domains and Relevant Criteria

**Figure 2:**
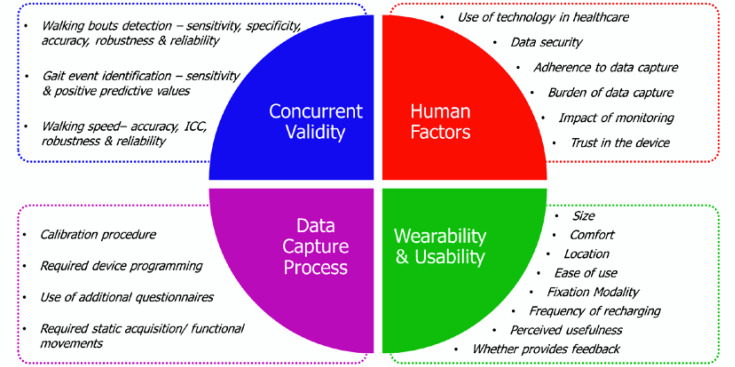
Identified key domains and their relevant criteria affecting wearable devices selection.

The factors to evaluate were grouped into the following four domains (d=1,…,4). Both domains and the associated criteria (c=1,…,N) were identified (Figure 2) through a combination of literature search and expert opinions within the IMI Mobilise-D consortium, which consists of many of the world’s leading scientists, clinicians, and companies on mobility assessment (>150 professionals from 34 partners; https://www.mobilise-d.eu):

*Concurrent validity*–factors related to the validity of the measurements;
*Human factors*–factors related to the context of data capture, perception of the user towards the technology, data security and privacy, effect of monitoring outside clinical settings;
*Wearability & usability for the wearer*–e.g., size, location, fixation modality, charging frequency;
*Data capture process*–e.g., whether a calibration procedure, device programming, or anthropometric information are required for appropriate data capture.

#### 2.1.1. Concurrent Validity criteria

To properly assess the criteria within this domain, reference parameters measured with a gold standard system (e.g., stereophotogrammetry or instrumented walking for mobility evaluation) need to be available. While several parameters can be captured during continuous mobility monitoring, this study focused on real-world walking speed (RWS), as a representative example. Level of agreement (expressed as the interclass correlation coefficient–ICC), accuracy, robustness, and reliability of RWS measurements can be assessed to quantify associated sources of error. Since the validity of RWS estimation depends on both the identification of a walking bout [5], and the initial and final contacts of the foot with the floor [29], the validity of these events needs to be considered as well.

#### 2.1.2. Human Factors Criteria

Acceptance and adoption of wearable devices are affected by the wearer’s view on the use of such devices to manage their health condition [33,34], data security [33], and their experience of, and adherence to, the proposed data capture process [38]. Of paramount importance for the wearer is the perceived impact that being monitored can have on daily life activities, as well as trust in the measurements collected by the device; perceived usefulness strongly correlates with wearer acceptance [43].

#### 2.1.3. Wearability and Usability Criteria

Widespread deployment of wearables requires “perceived usefulness” by the stakeholders, and benefits of use to be balanced with “perceived ease of use” [43]. Comfort, battery life, and feedback provided by the device are additional elements to be considered within this domain [35,36], as well as its size, location, and method of attachment to the body [39,44,45].

#### 2.1.4. Data Capture Process Criteria

Some devices/algorithms perform optimally when additional calibration procedures are performed, such as holding a static posture [7,31], device programming [46], or providing anthropometric measurements as an input [6,21]. These elements directly affect participant–device interaction and should be accounted for.

### 2.2. Weighting System

A questionnaire was designed to establish the relevance (i.e., weighting system) of the selected domains and criteria. Approval from the University of Sheffield Research Ethics Committee (Application 027211) was obtained for this study, and participants agreed to take part in the research by completing the anonymous online form. The online questionnaire was circulated among 34 partner institutions belonging to the Mobilise-D consortium, which consists of more than 150 professionals (e.g., scientists, clinicians, and companies) working on mobility assessment using wearable devices (www.mobilise-d.eu), and its external collaborators. Before widespread distribution, the ad-hoc questionnaire was pilot tested for both readability and data acquisition by using feedback from various professionals.

**Figure 3:**
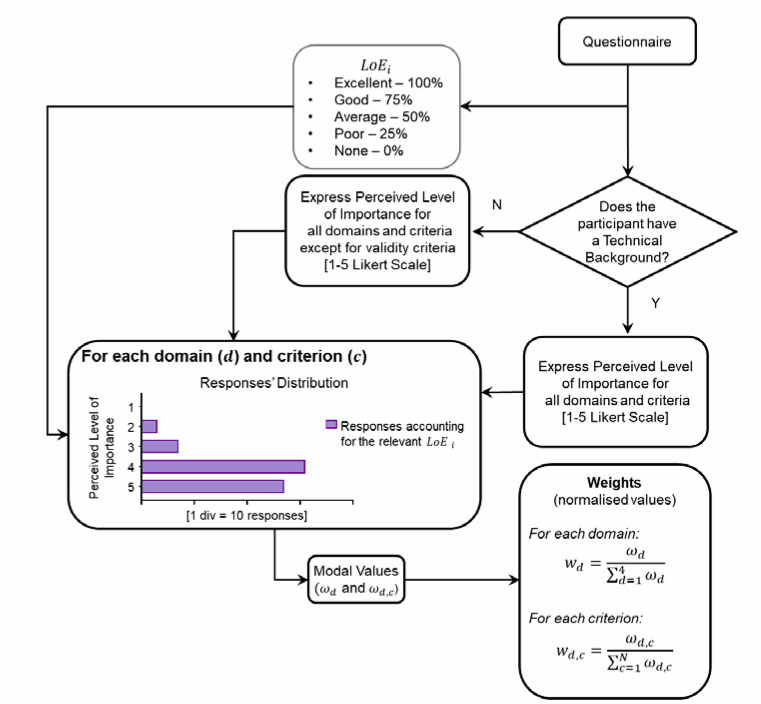
Process figure showing how the gathered responses about the perceived level of importance of the different domains and criteria are used to identify the normalized weights for each domain (*d*) and criterion (*c*).

Following the process visualized in Figure 3, the gathered responses were used to assess:

- Respondents’ background: clinical, technical, or both.
- Respondents’ level of expertise (LoE) with the use of wearable devices in clinical practice based on four questions:
  1. Do you know how a wearable device works and how it is used to identify gait features?
  2. As a researcher, have you ever used a wearable device?
  3. Have you ever used wearable devices directly on patients as opposed to healthy individuals?
  4. Have you ever analysed the information/data extracted from wearable devices to characterise patients’ mobility?
- Each positive response was scored as 0.25, and the total LoE was obtained as a sum of the partial scores. LoE of each participant was then classified as excellent, good, average, poor, or none if total LoE was 1.00, 0.75, 0.50, 0.25, and 0, respectively.
- Respondents’ perceived level of importance of each domain and criterion, based on a 1–5 Likert scale (1 = unimportant; 5 = very important).
- The modal value of the responses of each domain and criterion, $\omega_{d}$ and $\omega_{d,c}$, respectively, calculated as the preferences indicated by each respondent. The latter were multiplied by the relevant LoE, which allowed us to account for the relevant respondents’ level of expertise.

Finally, the computed weights were normalised as [42]:

(1) $$\mathrm{For}\;\mathrm{each}\;\mathrm{domain}\;d:\;w_{d}=\frac{\omega_{d}}{\sum_{d=1}^{4}\omega_{d}}$$

(2) $$\mathrm{For}\;\mathrm{each}\;\mathrm{criterion}\;c:\;w_{d,c}=\frac{\omega_{d,c}}{\sum_{c=1}^{N}\omega_{d,c}}$$

where N are the criteria included in the relevant domain (d).

### 2.3. Scoring System

Each criterion was first classified as either “benefit” or “cost” (Table 1) and scored higher/lower if implying a better/worse sensor/algorithm solution [42], using scores that were normalised concerning their range of variation within each criterion and domain:

(3) $$\mathrm{Benefit}\;\mathrm{criteria}\;e_{d,c}=\frac{s_{d,c}-\underset{i}{min}s_{d,c}}{\underset{d,c}{max}s_{d,c}-\underset{d,c}{min}s_{d,c}}$$

(4) $$\mathrm{Cos}t\;\mathrm{criteria}\;e_{d,c}=\frac{\underset{d,c}{max}s_{d,c}-\;s_{d,c}}{\underset{d,c}{max}s_{d,c}-\underset{d,c}{min}s_{d,c}}$$

where $s_{d,c}$ is the score assigned to the criteria c of the d domain ($0\leq e_{d,c}\leq 1$).

Only respondents who had declared to have a technical background were asked to score concurrent validity criteria based on the following definitions:

- *Accuracy*: closeness of an estimated parameter (p) to the “true value” measured using a gold standard ($p_{GS}$) and is expressed in percentage as:
  $$e\%=\frac{\left|p-p_{GS}\right|}{\left|p_{GS}\right|}\times 100$$
- *Robustness* to changes in the device positioning, quantified as e%.
- *Reliability* between different trials, quantified as e%.
- *ICC*: the agreement between p and $p_{GS}$ in different trials.
- *Sensitivity (%)*: describes the true positive (TP) events, i.e., the number of gait events (GEs–defined as initial and final foot-to-ground contacts and used to identify strides, steps, as well as gait cycle phases [18], expressed as unitless numbers) and Walking Bouts (WBs) correctly identified with a device/algorithm solution ($n_{GE}$) as compared to the values from a gold standard ($n_{GE\_GS}$):
  $$sens\%=\frac{n_{GE}}{n_{GE\_GS}}\times 100\;\mathrm{or}\;sens\%=\frac{TP}{TP+FN}\times 100$$
- *Specificity (%)*: number of true negative (TN) events relative to the actual events assessed with a gold standard:
  $$spec\%=\frac{TN}{FP+TN}\times 100$$
- *Positive predictive value (%)*: TP events over the total amount of identified GEs, including falsely detected GEs (TP+FP):
  $$PPV\%=\frac{TP}{TP+FP}\times 100$$

Criteria from the other domains were scored using the system shown in Table 1. Location and fixation modality criteria scores were defined by asking participants to rank possible choices taken from the literature [20,47,48,49]. They were then asked to indicate the best three from twelve locations (lower back/hip/waist; pocket; chest; neck (body-fixed); neck (pendant); head; foot; ankle; shank; thigh; wrist; arm) and five fixation modalities (adhesive on the skin; strap above/below clothes; clip above/below clothes). The recorded ranking scores (1, 2, 3 for 3rd, 2nd, 1st, respectively) were then scaled by the respondents’ LoE.

### 2.4. Comparison of Concurrent Solutions

For each monitoring solution ($E_{i}$), an overall score, based on the partial scores obtained for the different domains and criteria and on the calculated weights and scores, was finally computed:

(5) $$E_{i}=\overset{4}{\underset{d=1}{\sum }}e_{d}*w_{d}=\overset{4}{\underset{d=1}{\sum }}\left(\overset{N}{\underset{c=1}{\sum }}e_{d,c}*w_{d,c}\right)*w_{d}$$

where $e_{d}$ is the overall score of each domain d, obtained as the combination of the scores $e_{d,c}$ and normalised weights $w_{d,c}$, assigned to each of the N criteria.

### 2.5. Application of the Decision Matrix

Among the different studies in the literature evaluating either different solutions for DMOs estimations, the information and results extracted from two studies were used to feed the decision matrix and practically demonstrate how this tool can be used in future research.

1. Example 1. Three different concurrent methods [10,16,21] for gait temporal parameter estimations with a single device that was attached to the lower trunk [31].
2. Example 2. An evaluation of four (Movemonitor, Mc Roberts, The Hague, The Netherlands; Up, Jawbone, San Francisco, USA; One, Fitbit, San Francisco, USA; ActivPAL, PAL Technologies Ltd., Glasgow, UK) of the seven wearable devices placed in different locations as explored in Storm et al. [28].

Among the different domains’ proposed criteria, a subset of the available scores for the relevant studies was available and used in the decision matrix. The weighting systems were, therefore, accordingly adjusted based on the results obtained in this study. Benefit and cost scores were assigned based on Table 1 and the relevant information obtained through the ad-hoc questionnaire (i.e., fixation modality and device location) and normalised as described in Section 2.3. For each wearable device, the overall score was calculated using Equation (5).

## 3. Results

### 3.1. Participants

**Figure 4:**
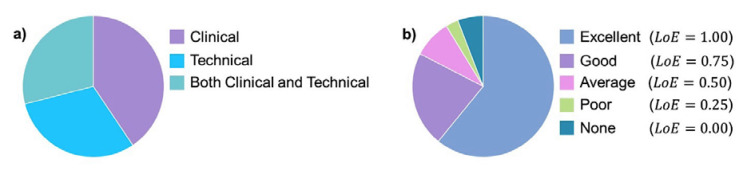
(**a**) Background of the respondents (*n* = 69); (**b**) Respondents’ level of expertise on the use of wearable devices in clinical settings as assessed through the purposely developed questionnaire.

Sixty-nine participants submitted their responses to the questionnaire (Figure 4). Among them, 83% had either an excellent or good level of expertise (LoE ≥ 0.75) in the use of wearable devices.

### 3.2. Weighting System

**Figure 5:**
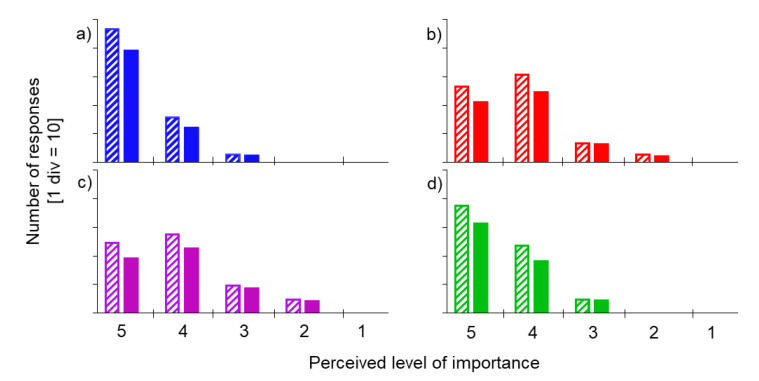
For each perceived level of importance (1–5 Likert scale; 1 = unimportant, 5 = very important), the absolute number of responses expressed by the participants for the four domains (**a**) concurrent validity, (**b**) human factors, (**c**) data capture process, and (**d**) wearability and usability) are shown with a pattern fill. The responses adjusted by the relevant LoE of each participant are shown with a solid fill.

Table 2 shows the normalised weights for domains and relevant criteria, as calculated based on each respondent’s perceived level of importance (Figure 5).

Based on the obtained modal values $\omega_{d}$ of each domain, both concurrent validity and wearability and usability domains were classified as “very important” for a seven-day mobility monitoring solution. The other two domains were labeled as “important”; and this classification was not modified when the respondents’ LoE was considered (Figure 5).

### 3.3. Scores

**Figure 6:**
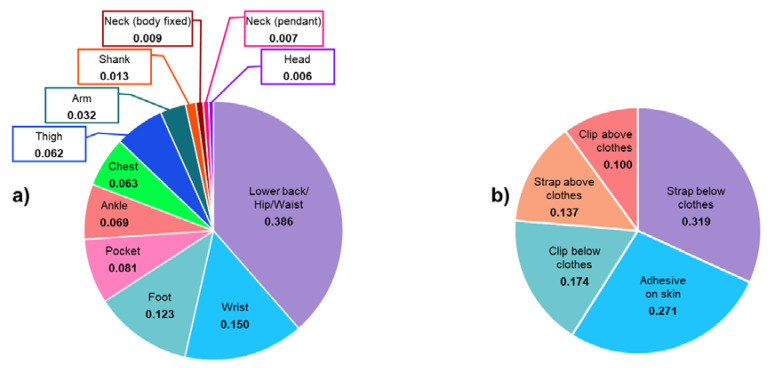
Scores for the different identified device locations (**a**) and fixation modality (**b**). Values were obtained based on the best three choices expressed from each participant and their relevant LoE.

The favourite location and fixation criteria were the “lower back/hip/waist” and “strap below clothes,” respectively, as shown by the results reported in Figure 6. The most common explanations behind the choice of the lower back/hip/waist location were the respondents’ previous experience with this solution with their patients, comfort, proximity to the centre of mass location, the possibility of the device to be integrated with a belt and the potential to “track” the movement of both lower limbs with a single device. The fixation with a strap below the clothes was indicated as preferred due to this method’s robustness, the possibility of hiding the sensor, and preserving participant privacy and past positive experiences with this approach.

### 3.4. Use of the Decision Matrix

#### 3.4.1. Example 1

Among the methods described in Reference [31], the three for which the robustness had been assessed (T1–Zijlstra and Hof [21]; T2–González et al. [10], T3–McCamley et al. [16], Table 3) were considered for the concurrent evaluation. Step time accuracy and robustness (highest e% value reported for each method) were considered as representative for walking bout detection accuracy and robustness (Table 3), respectively.

#### 3.4.2. Example 2

Among the seven wearable devices explored in Storm et al. [28], four (S1–Movemonitor, S2–Up, S3–One, S4–ActivPAL, Table 4) were selected for the concurrent evaluation, performed using step detection accuracy as a concurrent validity criterion (Table 4). The mean step detection accuracy value was calculated for each monitoring solution over those reported for slow, self-selected, and fast walking speeds.

## 4. Discussion

This study aimed to propose a standardised methodology for selecting the optimal device for continuous mobility monitoring, with a special focus on walking speed. Although this method was implemented using professionals/researchers, a similar approach could also be used to evaluate user perspectives. This approach’s novelty allows researchers to assess the relevance of domains that were previously quantified only in isolation [33,34,35,36,37,39,40], such as the wearability and usability of a device, in combination with aspects related to its validity and other domains. This ensures a more robust choice of a specific solution.

The different aspects to be considered while exploring concurrent continuous mobility monitoring solutions were first identified, and their relevance assessed by capturing information from experts in this research area. The identified domains of relevant criteria, and calculated weighting and scoring systems, were the three elements that identify the decision matrix, representing the successfully developed method.

The scoring system, which combined “benefit” and “cost” criteria, highlighted the differences among monitoring solutions and allowed the calculation of an overall score for each of them [42]. This procedure allows a trade-off on multiple and concurrent domain/criteria.

The weighting system was obtained via an experts’ questionnaire and constituted an objective methodology to assess the selected elements’ relevance while aiming to identify an optimal monitoring solution. Critically, this method’s reliability does not rely on the knowledge and expertise of a single decision-maker, which could bias the outcomes [42]. The novelty of this developed approach is that it allows researchers to consider the respondents’ expertise, making the unbiased results especially relevant for the field. The use of examples taken from the literature demonstrated how this framework could be used when only a subset of domains/criteria are available by adjusting the relevant scoring system to specific requirements.

From a professional’s perspective, the concurrent validity domain, which is the one most widely considered in the literature when a new wearable device is proposed, was also confirmed to be the most important in this study (37%), even by respondents from a non-technical background (33%). Nonetheless, results indicate that the other domains are also important for the widespread deployment of wearable devices (wearability and usability: 30%; human factors: 17%; data capture process: 16%). Recently, a study [50] attempted to provide some guidelines for selecting and comparing different devices; however, the focus was still mostly on how technical specifications and raw data quality affect the validity domain.

Respondents to the questionnaire, who were professionals (i.e., developers, clinicians, and researchers) who deploy the technology, were asked to select the best three location and fixation solutions. This has allowed for the establishment of an exact ranking among different solutions for continuous mobility monitoring. Although previous studies have assessed the effect of different device locations [23,24,25,26,27,28,29,30,31,32], the effect of a variety of fixation methods had not yet been explored. Thus, we have developed and applied a novel quantitative approach to allow these criteria to be explicitly identified and ranked. Almost 90% of the responders chose a device placed on the lower back (of these, 62%, 24%, and 14% identified this as the first, second, and third choice, respectively) because it provides accurate measurement and can be integrated with a belt. This solution is indeed usually accepted for long-term at-home use, approximates the centre of mass location, and is the most common location adopted in studies assessing mobility [48]. For the fixation method, the solution identified as an ideal one can be hidden under the clothes (83% of the respondents). In particular, a strap (43%) and adhesive on the skin (43%) were indicated as the most robust fixation methods (i.e., less relative motion between the device and the segment where it is placed). Other explicit preferences for location and fixation modality included choosing a solution that provides reliable measures, allows comfort, as well as device aesthetics, confirming what has been previously reported in the literature [35,43].

Once developed, the proposed framework was successfully used for ranking concurrent solutions using data extracted from the literature to compare different algorithms applied to the same raw data, which led to conclusions similar to those from the original study [31] while also providing a single summary score for each proposed solution. When used to evaluate the performance of the different wearable devices reported in Storm et al. [28], the differences among the solutions can be further highlighted, not just considering their concurrent validity, but also the other three domains, which are key elements for the widespread use of this technology. Moreover, a similar methodology could also be implemented when selecting concurrent devices for different applications.

Users (i.e., wearer, either patients or participants), which are the real stakeholders who will directly use this technology, did not participate in this questionnaire, which certainly represents the main limitation of this study. Nonetheless, their opinion might have been biased by their previous experience, which is usually limited to using a single wearable device. In order to include this essential aspect, future studies should either recruit a specific population of individuals who previously experienced different wearable solutions for mobility monitoring or having them participating in an ad-hoc comparative assessment. As highlighted by Manta et al. [51], both patients and care partners should be engaged in the selection and development of digital mobility outcome solutions for identifying a solution that is effective, helpful, and improves both quality and efficiency in clinical research and care. Future studies might include the opinion from a specific population of individuals who previously experienced different wearable solutions for mobility monitoring or include them in a comparative assessment. Their relevant perception of the importance of the identified factors could then be integrated and combined with the information collected in this study. Moreover, it would be of interest to evaluate the effect that awareness of the criteria adopted behind the design of a device might have on user perception and acceptance of the device.

## 5. Conclusions

This study proposed a new methodology that provides a novel, holistic, objective, as well as standardised approach accounting for complementary aspects that should be considered by professionals and researchers when selecting a solution for continuous mobility monitoring. An ad-hoc decision matrix has been established for this aim, the definition of which made it explicit that a comprehensive approach should be adopted when choosing a technology for continuous mobility monitoring if aiming for widespread adoption. In particular, the four identified domains: concurrent validity, human factors, wearability and usability, and data capture process should be simultaneously considered when evaluating concurrent solutions.

## Figures and Tables

**Table 1:** Cost/benefit criteria and scoring system.

Domain	Criterion	Benefit	Cost	Score
Concurrent Validity	Walking speed accuracy		✓	Scores based on the relevant technical definitions
	Walking speed robustness		✓	
	Walking speed reliability		✓	
	Walking speed–Interclass coefficient	✓		
	Walking bout detection sensitivity	✓		
	Walking bout detection specificity	✓		
	Walking bout detection accuracy		✓	
	Walking bout detection robustness		✓	
	Walking bout detection reliability		✓	
	Gait event sensitivity	✓		
	Gait events identification	✓		
Human Factors	Use of technology in healthcare *	✓		–
	Data security	✓		Yes(1)/No(0)
	Adherence to data capture	✓		Yes(1)/No(0)
	Burden of data capture *		✓	–
	Impact of monitoring		✓	Yes(1)/No(0)
	Trust in the device	✓		Commercial: Yes(1)/No(0)
Wearability and usability	Comfort *	✓		–
	Location	✓		^1^
	Ease of use		✓	Interaction: Yes(1)/No(0)
	Frequency of recharging		✓	Battery Life ^2^
	Perceived usefulness *	✓		NA
	Whether it provides feedback	✓		Yes(1)/No(0)
	Size		✓	width x height x depth x mass
	Fixation modality	✓		^1^
Data Capture Process	Calibration procedure		✓	Yes(1)/No(0)
	Required static/functional movements		✓	Yes(1)/No(0)
	Required device programming		✓	Yes(1)/No(0)
	Questionnaires/Anthropometric measures		✓	Yes(1)/No(0)

^1^ Scores established via the purposely developed questionnaire. ^2^ Daily recharging (5/5); 2–3 days BL (4/5); 4–5 days BL (3/5); 6–7 days BL (2/5); 7+ days BL (1/5). * Scores usually established through dedicated questionnaires available in the litarature.

**Table 2:** Weighting system.

Domains		Criteria	
	Weight		Weight
Concurrent Validity	0.368	Walking speed accuracy	0.133
		Walking speed reliability	0.130
		Walking speed robustness	0.107
		Walking speed–Interclass coefficient	0.107
		Walking bout detection specificity	0.097
		Walking bout detection reliability	0.095
		Walking bout detection accuracy	0.087
		Walking bout detection sensitivity	0.064
		Walking bout detection robustness	0.062
		Gait event sensitivity	0.059
		Gait events identification (PPV)	0.057
Human Factors	0.175	Trust in the device	0.193
		Burden of data capture	0.193
		Data security	0.181
		Impact of monitoring	0.163
		Adherence to data capture	0.136
		Use of technology in healthcare	0.134
Wearability and usability	0.296	Ease of use	0.185
		Comfort	0.168
		Fixation modality	0.141
		Size	0.119
		Location	0.116
		Perceived usefulness	0.096
		Frequency of recharging	0.092
		Whether it provides feedback	0.083
Data Capture Process	0.161	Calibration procedure	0.326
		Required static/functional movements	0.286
		Required device programming	0.197
		Questionnaires/Anthropometric measures	0.192

**Table 3:** Evaluation matrix applied to three concurrent methods. Normalised scores are reported in bold.

Domains		Criteria				
	Weight		Weight	T1	T2	T3
Concurrent Validity	**0.368**	Walking bout detection accuracy ^1^	0.328	8	4	2
				**0.00**	**0.67**	**1.00**
		Walking bout detection robustness ^1^	0.234	9	4	2
				**0.00**	**0.71**	**1.00**
		Gait event identification (PPV)	0.215	100	97	100
				**1.00**	**0.00**	**1.00**
		Gait events sensitivity	0.223	97	82	100
				**0.83**	**0.00**	**1.00**
Human Factors	**0.175**	Trust in the device	0.516	1	1	1
				**1**	**1**	**1**
		Data security	0.484	1	1	1
				**1**	**1**	**1**
Wearability & usability	**0.296**	Fixation modality	0.301	0.137	0.137	0.137
				**1.00**	**1.00**	**1.00**
		Size	0.254	525.76	525.76	525.76
				**1.00**	**1.00**	**1.00**
		Location	0.248	0.386	0.386	0.386
				**1.00**	**1.00**	**1.00**
		Frequency of recharging	0.197	1	1	1
				**1.00**	**1.00**	**1.00**
Data Capture Process	**0.161**	Calibration procedure	0.326	0	0	0
				**1.00**	**1.00**	**1.00**
		Required static/functional movements	0.286	1	1	1
				**0.00**	**0.00**	**0.00**
		Required device programming	0.197	0	0	0
				**1.00**	**1.00**	**1.00**
		Questionnaires/Anthropometric measures	0.192	1	0	0
				**0.00**	**1.00**	**1.00**
			**Overall score**	**0.70**	**0.73**	**0.95**

^1^ Represented as step time accuracy and robustness.

**Table 4:** Evaluation matrix applied to four wearable devices. Normalised scores are reported in bold.

Domains		Criteria					
	Weight		Weight	S1	S2	S3	S4
Concurrent Validity	**0.368**	Step detection accuracy	1.000	1.483	4.897	1.567	2.493
				**1.00**	**0.00**	**0.98**	**0.70**
Human Factors	**0.175**	Trust in the device	0.516	1	1	1	1
				**1.00**	**1.00**	**1.00**	**1.00**
		Data security	0.484	1	1	1	1
				**1.00**	**1.00**	**1.00**	**1.00**
Wearability & usability	**0.296**	Fixation modality	0.301	0.319	0.174	0.174	0.271
				**1.00**	**0.00**	**0.00**	**0.67**
		Size	0.254	3910.62	23.17	79.01	259.70
				**0.00**	**1.00**	**0.99**	**0.94**
		Location	0.248	0.386	0.15	0.386	0.013
				**1.00**	**0.37**	**1.00**	**0.00**
		Frequency of recharging	0.197	0.2	0.2	0.2	0.2
				**1.00**	**1.00**	**1.00**	**1.00**
Data Capture Process	**0.161**	Calibration procedure	0.326	0	0	0	0
				**1.00**	**1.00**	**1.00**	**1.00**
		Required static/functional movements	0.286	0	0	0	0
				**1.00**	**1.00**	**1.00**	**1.00**
		Required device programming	0.197	1	0	0	0
				**0.00**	**1.00**	**1.00**	**1.00**
		Questionnaires/Anthropometric measures	0.192	0	0	0	0
				**1.00**	**1.00**	**1.00**	**1.00**
			**Overall score**	**0.89**	**0.41**	**0.81**	**0.78**
